# Uncovering marine connectivity through sea surface temperature

**DOI:** 10.1038/s41598-021-87711-z

**Published:** 2021-04-23

**Authors:** Ljuba Novi, Annalisa Bracco, Fabrizio Falasca

**Affiliations:** 1 Institute of Geosciences and Earth Resources (IGG), National Research Council (CNR), 56124 Pisa, Italy; 2 School of Earth and Atmospheric Sciences, Georgia Institute of Technology, Atlanta, GA USA

**Keywords:** Marine biology, Biogeography, Biooceanography, Ecological networks, Physical oceanography

## Abstract

A foundational paradigm in marine ecology is that Oceans are divided into distinct ecoregions demarking unique assemblages of species where the characteristics of water masses, and quantity and quality of environmental resources are generally similar. In most of the world Ocean, defining these ecoregions is complicated by data sparseness away of coastal areas and by the large-scale dispersal potential of ocean currents. Furthermore, ocean currents and water characteristics change in space and time on scales pertinent to the transitions of biological communities, and predictions of community susceptibility to these changes remain elusive. Given recent advances in data availability from satellite observations that are indirectly related to ocean currents, we are now poised to define ecoregions that meaningfully delimit marine biological communities based on their connectivity and to follow their evolution over time. Through a time-dependent complex network framework applied to a thirty-year long dataset of sea surface temperatures over the Mediterranean Sea, we provide compelling evidence that ocean ecoregionalization based on connectivity can be achieved at spatial and time scales relevant to conservation management and planning.

## Introduction

In marine realms, the identification of areas with relatively homogeneous ecosystems (ecoregionalization) and their connectivity is key to environmental management and species conservation^1^. These ecoregions provide a framework for investigating complex problems, from monitoring pollutant or invasive species dispersal to designating effective marine protected areas^2,3^. The potential of a marine protected area to retain biodiversity and restock species abundance beyond its border, for example, is inherently linked to some form of regional discretization and population connectivity, such as larval and juvenile dispersal or seeding across ecoregions^4^.

The intrinsic complexity of the processes and scales involved makes marine ecoregionalization challenging. Different methodologies have been proposed so far: (1) the taxonomic approach^5–7^ ,(2) the ecological approach^8–10^, (3) the integrated taxo-ecological approach^11^, and (4) the connectivity-based approach^2,12–16^. Among these, (1) relies on species distributions and classifies regions according to similar aggregations of species; (2) identifies areas by common cycles of biogeochemical or physical properties; and (3) adopts a combination in which both the environment and the species are accounted for altogether. These three methodologies share a limiting factor: they all omit the effects of oceanic currents on larval dispersal and on connectivity or isolation between ecoregions. This problem is overcome by the connectivity-based approach (4), where dispersal processes are identified through Lagrangian methods, at times coupled to network approaches^14^. The need to account for dispersal is motivated by the metapopulation concept^17,18^: the distribution of marine species is not solely driven by local conditions, but also by the spreading of propagules, larvae, juveniles, as well as adult organisms.

As ocean circulation models and reanalysis datasets have become more readily available and reliable, the connectivity-based ecoregionalization has been attempted for some basins^2,19–21^. However, simulating larval dispersal using Lagrangian particle tracking is a computationally intensive task. For example, in the work of^2^ Lagrangian particles were regularly seeded every 3 days and 10 km at three different depths (0.5 m, 50 m, 100 m) over the entire Mediterranean Sea. The particles were advected by the velocity field from an ocean model run at 1/12° horizontal resolution and the ecoregions were then calculated through a hierarchical clustering applied to a distance matrix. Computational requirements constrained this work to a period of 4 years. Eco-provinces from Lagrangian trajectories have been determined also using flow networks^14^ adapting the *infomap* community detection algorithm^16^. In these brute-force, Lagrangian-based approaches, the need for large spatial coverage dramatically reduces the maximum time span and vice versa.

To date, no ecoregionalization method informs scientists and stakeholders about ecosystem connectivity at the basin scales and interannual to decadal times toward which management measures and marine protected area designations should aspire. We propose to this end a complex networks-based framework (“δ-MAPS”^22,23^) applied to monthly sea surface temperature (SST) anomalies for ecoregionalization and connectivity inference.

δ-MAPS has the advantage, compared to most other network methods, of identifying spatially contiguous, functional domains. The use of SST anomalies is justified by the dynamic link that relates them to sea surface height on small spatial scales (order of 100 km, the so-called mesoscales) and low temporal frequencies (monthly and longer)^24^, which are the ones of interest to basin-scale marine ecosystem connectivity. In the search for a variable that is easily observable with a uniform coverage, linked to the flow advective properties, and containing as much useful information about habitability for a given species as possible, the SST field satisfies all criteria: It is observable through satellite platforms; at equatorial, tropical and mid-latitudes its anomalies are strongly coupled with the layers underlying the ocean surface through horizontal oceanic currents and mixing; and it modulates habitability directly, as well as indirectly, through the solubility of oxygen and carbon dioxide.

The modulation of SST anomalies by ocean surface currents and horizontal advection is verified as we test the new framework using a recently developed reanalysis for the Mediterranean Sea that spans 30 years at spatial resolution of 6–7 km (CMEMS MED-Physics^25^) but in future application satellite products could be used instead. The choice of the Mediterranean Sea is motivated by the existence of previous works that we can use for validation purposes, and by the urgency of establishing better management protocols in this basin.

**Figure 1:**
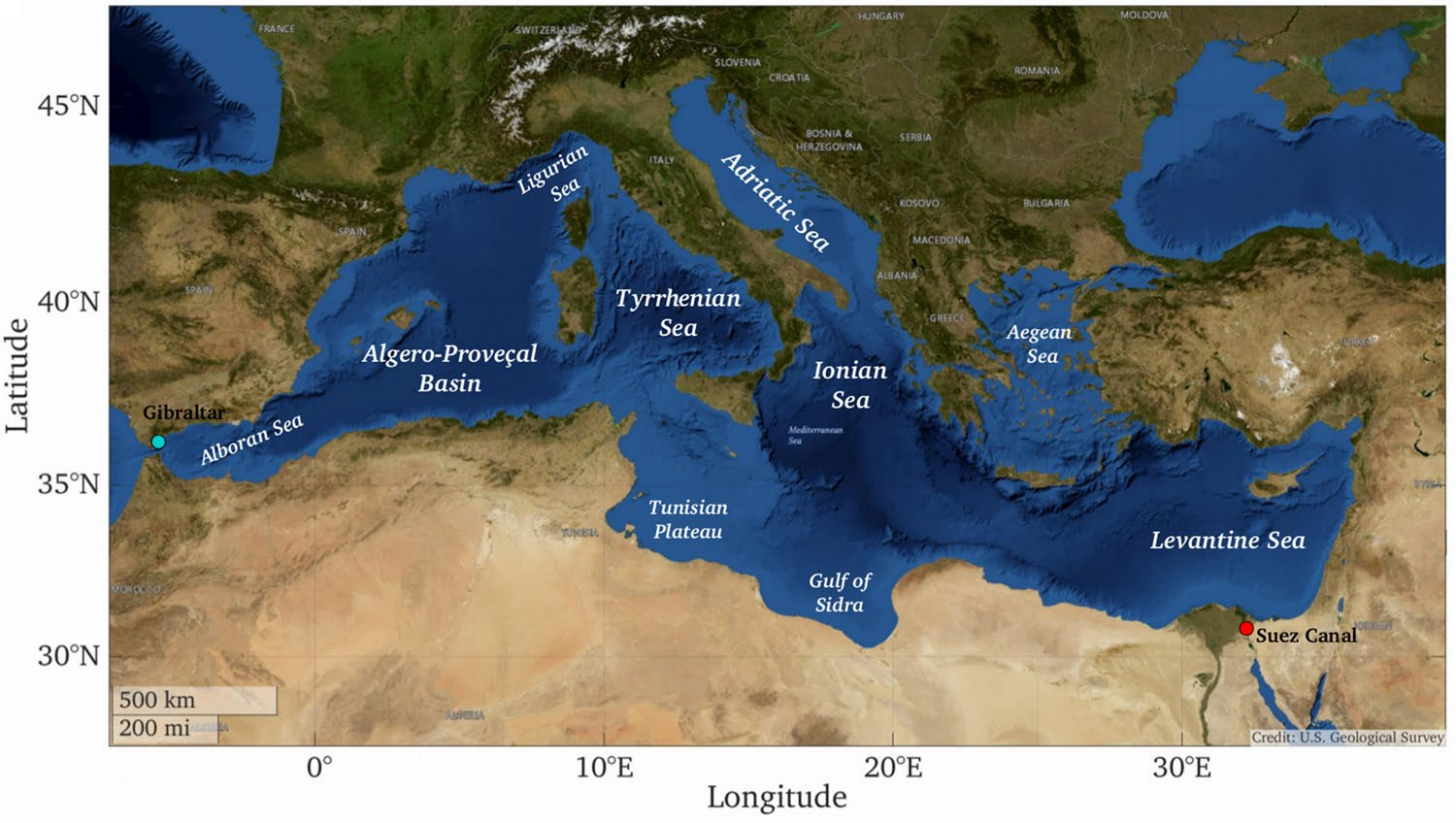
The Mediterranean Sea and its sub-basins. The red dot indicates the Suez Canal, which physically connects the Red Sea with the Levantine Sea; the green dot shows the Gibraltar Strait location, from which Atlantic waters enter the Mediterranean Sea near the surface, and vice versa at depth. (USGS Imagery Topo: courtesy of the U.S. Geological Survey. Map visualization produced with Matlab R2018a, https://www.mathworks.com/).

The Mediterranean Sea is a critical biodiversity hotspot threatened by climate change. It covers a small fraction of the global ocean surface area (0.82%) and volume (0.32%)^26^ but hosts roughly 17,000 species, retaining a large endemism^26–28^. The general circulation in the Mediterranean Sea, together with its latitudinal extent, seasonal cycle, and complex bathymetry, allows wide environmental and biological diversity across scales^26^. The near surface circulation consists of separate anticyclonic gyres in the western and eastern portions, connected through the Strait of Sicily (Fig. 1). The complexity of its coastlines, especially on the northern side, and the presence of numerous islands promote the formation of eddies, fronts and other local persistent currents. The overall hydrography^28–30^, as well as its general oligotrophy^2,28^, creates heterogeneous ecosystem conditions and functional habitats. In the past decades, the area of increased sea water temperatures have expanded from the southern and eastern portion of the basin into the northward and westward direction, threatening local habitats and productivity. Together with warmer temperatures, changes in biodiversity have been recorded following the invasion of alien species especially in the eastern basin where it has been fostered by the opening of the Suez Canal^28,31^. In light of these trends and of the overall role of the Mediterranean basin in the global marine biodiversity, it is worthwhile to test the proposed methodology to explore long-term changes in its ecoregionalization.

## Methods

### δ-MAPS

δ-MAPS aims at reducing a generic spatiotemporal field ***X***(t) dimensionality by identifying spatially contiguous regions (referred to as *domains*) and their connectivity patterns. A domain is a spatially contiguous region in a given field that participates in the same dynamic functions, such that grid cells belonging to that domain share a highly correlated temporal activity. Links between domains define a functional network with a weight assigned to each edge to reflect the magnitude of interaction between any two domains. The strength of a domain is then defined as the sum of the absolute weights of all edges. It is therefore an ideal tool to identify ecoregions if applied to a field that informs us of the connectivity properties of the flow.

*δ*-MAPS works through two steps, namely domain identification and network inference, and we refer the reader to Ref.^22^ for a detailed description.

### Domain identification

Given a field ***X***(t), its dimensionality is reduced by identifying sets of grid cells (i.e., domains) with highly correlated activity. To do so it is hypothesized that domains have *epicenters* or *cores*, where their local homogeneity is greatest, and these cores are identified. Formally, each grid cell *i* is associated with a time series *x*_*i*_(t), after removing a linear trend and seasonality, and for each *i* we define a *K*-neighborhood $\gamma_{K} \left( i \right)$ including the grid cell *i* and its *K* closest neighbors. The local homogeneity of *i* is then computed as the average pairwise correlation in its $\gamma_{K} \left( i \right)$. A grid cell is a core if its local homogeneity is a local maximum and greater than a threshold δ. Cores are then expanded and merged to identify domains.

### Network inference

For each domain *A*, we compute its signal $X_{A} \left( t \right)$ as the weighted cumulative anomaly of all time series within it:

1 $$X_{A} \left( t \right) = \mathop \sum \limits_{i = 1}^{\left| A \right|} x_{i} \left( t \right)\cos \phi_{i} ,$$

where $x_{i} \left( t \right)$ is a time series of length $T$ associated to grid cell $i$ with latitude $\phi_{i}$ and $\left| A \right|$ is the number of grid cells in *A*.

Given D domains, the network is inferred by considering each possible pair of domains *A* and *B* and computing their Pearson correlation ${\text{r}}_{A,B} \left( \tau \right)$ for a lag range $\tau \in \left[ { - \tau_{\max } ,\tau_{\max } } \right]$. Given a significance level, the significance of each pairwise correlation is tested excluding autocorrelations using the Bartlett’s formula^42^.

Two domains *A* and *B* are connected if there exists at least one significant correlation between the two at any lag in the range $\tau \in \left[ { - \tau_{\max } ,\tau_{\max } } \right]$, denoted as *R*_*A,B*_* (τ).* If *R*_*A,B*_* (τ)* includes the lag $\tau = 0$, the link is left undirected. If *R*_*A,B*_* (τ)* is strictly positive (negative) the link will be directed from *A* to *B* (*B* to *A*). A weight $w_{A,B}$ is assigned to each link based on the covariance between the two signals $X_{A} \left( t \right)$ and $X_{B} \left( t \right)$ at the lag $\tau^{*}$ at which their significant correlation $r_{A,B} \left( {\tau } \right)$ is maximized. Finally, a (non-dimensional) strength value is assigned at each domain, defined as the sum of the absolute weights of all the links connected to that domain.

In this work, δ-MAPS is applied to the Mediterranean Sea Physical Reanalysis (CMEMS MED-Physics^25^), which is the result of the Mediterranean Forecasting System run with horizontal grid resolution of 1/16° (ca. 6–7 km) complemented by a variational data assimilation scheme (OceanVAR) for temperature and salinity vertical profiles and satellite sea level anomaly along track data. The significance level for the network inference is set to 0.03 and tested using a t-test. We used *τ*_*max*_ = 12 months and a *K-*neighborhood of 4 grid cells. The δ threshold (see Ref.^22^) is inferred using a statistical significance level α. In the validation test over 2007–2010 (see “Results”) α = 10^–3^, in the networks built using 7-year time slots α = 2 × 10^–5^, and in those using 6 and 8 years α = 6 × 10^–5^ and α = 3 × 10^–6^, respectively (see “Results” for timeslots definition). Different values of α are set in order to maintain the minimum significant correlation for the domain identification within a fixed range (0.54–0.55) for the time slots containing the validation period, independently of the time slots considered. This guarantees that ecoregions are consistent with those obtained over the validation period.

## Results

The *δ*-MAPS analysis is performed onto monthly mean SST anomalies from the Mediterranean Sea Physical Reanalysis (CMEMS MED-Physics^25^) over the period 1987–2017. The advantage of using a reanalysis resides in the availability of a velocity field consistent with the SSTs that allows us to confirm the coupling between network domains and ocean currents within the euphotic layer.

### Validation of the *δ*-MAPS framework

The proposed ecoregionalization is first applied to the 2007–2010 period, when the *domains,* representing ecoregions, can be compared to those identified by Ref.^2^ using Lagrangian methods. The details of this validation are reported below and relevant figures can be found in the *Supplementary Information*.

The 2007–2010 ecoregions in Figure S1 are consistent with the ones derived in Ref.^2^ through computationally intensive simulations. The name of each domain corresponds with those used in Ref.^2^ to ease comparison. It is worthwhile remarking that this work and the one of Ref.^2^ not only use very different methods to define connectivity, but also different data sources. Our study uses velocity and SST output fields from CMEMS MED Physics reanalysis, while Ref.^2^ uses the configuration PSY2V3 of the operational system MERCATOR OCEAN with a resolution of 8 km in the horizontal downscaled to a connectivity grid of 50 × 50 km. The data assimilation and clustering algorithms are different and Ref.^2^ employs a cut-off in addition to the clustering grid downscaling. These differences unavoidably translate into slightly different shapes and patterns of the domains inferred. For example, the D + V area in panel (a) of Figure S1 is effectively two separate ecoregions in Ref.^2^, in which the Messina Strait is not resolved at the connectivity grid level. However, this separation appears inconsistent with the surface kinetic energy (K.E.) of panel (b) in Figure S1, computed from the horizontal currents, e.g. zonal (*u*) and meridional (*v*) velocity components, as *K.E.* = *1/2 |V|*^*2*^ where *|V|*= *(u*^*2*^ + *v*^*2*^*)*^*0.5*^. Indeed, there is no clear separation between the regions north and south at Messina Strait in our dataset. Having detailed this example and acknowledged that some differences should be expected, the overall basin eco-regionalization using *δ*-MAPS is consistent with that in Ref.^2^. The spatial accuracy is enough to well separate the main ecological areas, despite small-scale differences (i.e. some km, due to resolution choices).

By and large, the SST anomaly domains in Figure S1 are bounded by ocean currents, in agreement with Ref.^2^. This is due to the dominance of advective forcing by ocean currents on the SSTs at equatorial and mid latitudes, on monthly timescales and spatial scales of few hundreds kilometers^24^. This link, which is foundational to the proposed methodology, is further quantified as follows: First, we calculate the surface K.E. per unit mass averaged over the time slot of interest; second, we select the points in the validation period (2007–2010) that exceed the 50^th^ percentile of surface K.E. computed for the entire basin over 1987–2017 (e.g. 0.004 m^2^/s^2^); third, we compute the domain-boundary matrix augmented by 1 grid point in each direction; finally, we count which fraction of the domain boundaries computed in the boundary matrix overlaps with the K.E. fronts (above the 50th percentile threshold). The fraction obtained is high and equal to 0.73, and remains elevated when increasing the threshold to the 60th percentile (0.66). This procedure was repeated for all the time slots with Δ = 7 years used next in this study*,* obtaining high and very stable values in each case (mean ± variance = 0.73 ± 0.01 for the 50th percentile threshold, and 0.65 ± 0.01 for the 60th percentile threshold).

Additionally, the correlation between the surface K.E. and K.E. at 50 m, 100 m, and 150 m over the whole 1987–2017 period (Figure S2) remains positive and significant, with coefficients for the whole domain (field mean c.c.  ± variance) of 0.83 ± 0.04 at 50 m, 0.68 ± 0.05 at 100 m, and 0.54 ± 0.06 at 150 m, indicating that the link extends to the whole euphotic layer.

### Mediterranean Sea ecoregions: long-term changes

**Figure 2:**
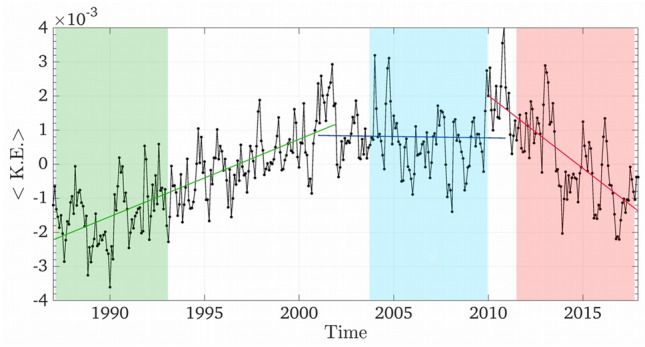
Mean surface kinetic energy timeseries. Monthly time series of deseasonalized surface kinetic energy per unit mass (m^2^/s^2^), averaged over the whole Mediterranean Sea between 1987 and 2017. The shaded areas indicate the 1987–1993 (during the UP period), 2004–2010 (during MAX) and 2011–2017 (during DOWN) time slots used in Fig. 3.

The space-averaged (e.g. averaged on the whole basin) SSTs over the 1987–2017 period are characterized by a linear warming trend of about 0.04 °C per year, stronger in the eastern portion of the basin (Figure S3 in *Supplementary Information*). Over the same period, the K.E. per unit mass is characterized by different trends over decadal or quasi-decadal periods (Fig. 2, shown for surface only but the trend extends similarly to 50 m and 100 m depths) and no clear east–west contrast. A positive trend is found in the first part of the curve (1987–2001, 2.3 × 10^–4^ m^2^/s^2^ per year, green line in figure), followed by a central decade without statistically significant changes (2001–2010, blue line), and a steep negative trend afterward (2010–2017, – 4.1 10^–4^ m^2^/s^2^ per year, red line). We refer to 1987–2001, 2001–2010 and 2010–2017, as the *UP*, *MAX* and *DOWN* periods. The dynamical changes associated with the strengthening and weakening of ocean currents are hypothesized to coincide with a reshaping of the sub-basin ecoregions and reciprocal connectivity. The ecoregionalization inference is therefore performed considering time slots of varying length, so that *yr*_*end*_ = *yr*_*ini*_ + *Δ* with *yr*_*ini*_ = *y*_*0*_ + *n, n* = *0,1,…,N,* where *y*_*0*_ is the initial year of the dataset (1987) and N is the total number of time slots, each of duration *Δ* years, between 6 and 8. Time slots overlapping by more than one year among different trends periods are excluded. The choice of Δ = 7 years represents the best trade-off for having enough time slots to quantify the evolution of ecoregions and a sufficiently large number of data points in each time slot for statistical inference. We will focus on this case, but results are verified also for the other *Δ* values (see *Supplementary Information*).

**Figure 3:**
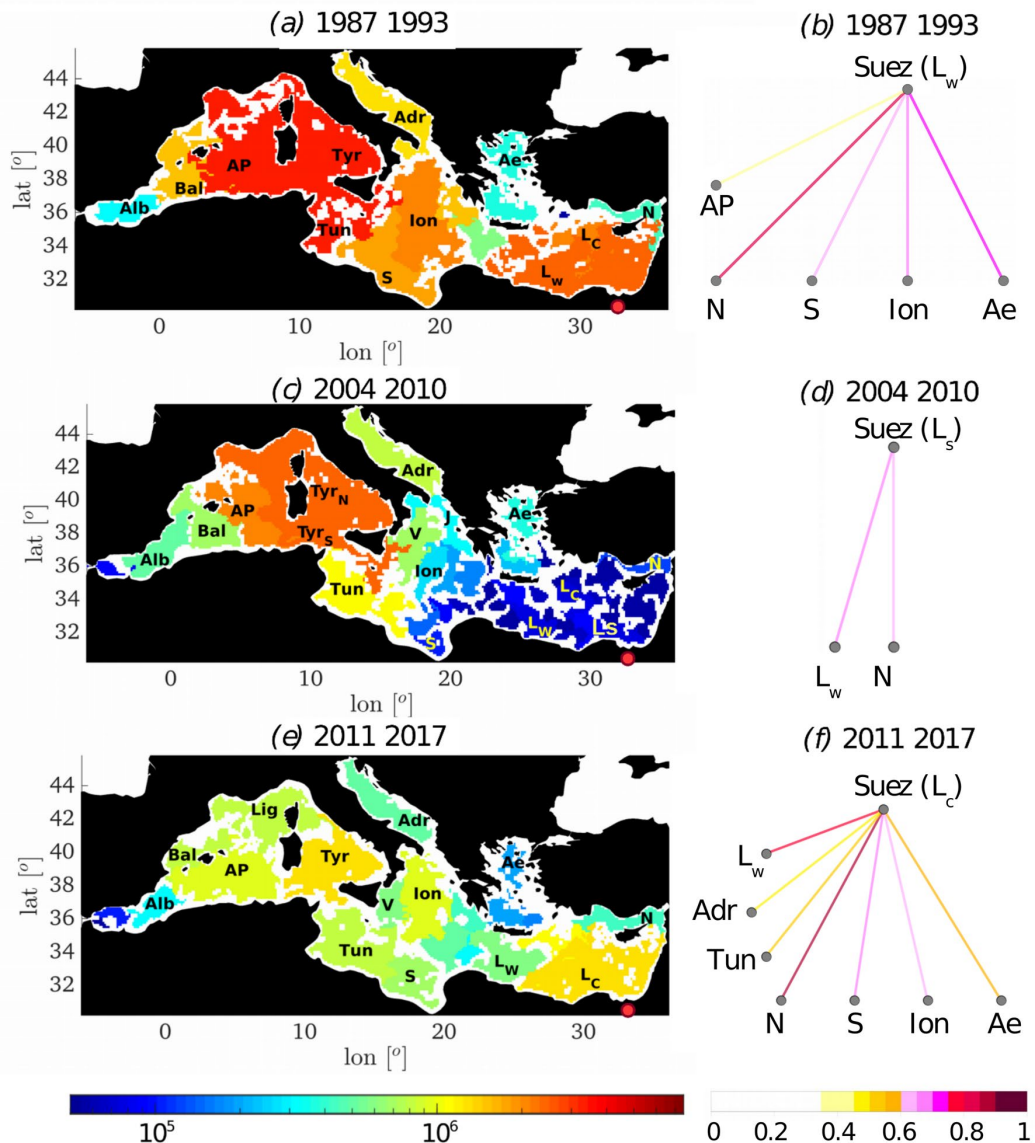
Domains and connectivity networks for the domain containing the Suez Canal. The three 7-year timeslots selected as representative of the UP (**a**,**b**), MAX (**c**,**d**) and DOWN (**e**,**f**) periods. The color of the domains represents their strength (left column), and the red dot shows the location of the Suez Canal. Links in the connectivity nets (right column) are colored according to the correlation between (the domain containing) the Suez Canal and other domains as labeled. Only correlations stronger than 0.35 are plotted. (Domains maps visualization produced with Matlab R2018a, https://www.mathworks.com/).

Strength maps for three representative time slots are presented in Fig. 3a,c,e while maps of domain strengths for all Δ = 7 time slots can be found in Figure S4. The mean surface kinetic energy averaged within each timeslot is next compared to the number of ecoregions in corresponding timeslots. The fragmentation level, or the total number of ecoregions, and the mean surface kinetic energy content are highly correlated (Figure S5b in *Supplementary Information*), with a Pearson’s coefficient of 0.79 for the whole Mediterranean Sea, and 0.8 (0.65) for the eastern (western) basin. The fact that time slots are not independent does not invalidate the analysis, and a large correlation (c.c = 0.73) is retained even when using four non-overlapping time slots. A higher fragmentation occurs whenever the upper ocean layer is more energetic, and this relationship is robust to changes of *Δ* (see *Supplementary Information*). The domain strength is next compared to the mean K.E. content. For each timeslot, the domain strength is spatially averaged over the eastern and western basin separately. The correlations between the averaged strengths and the corresponding time slot mean surface K.E. values, both varying as the time slots change, are then calculated for eastern and western basins separately. No linkage is found in the western basin, but a strong anticorrelation describes the relationship in the eastern Mediterranean (c.c. − 0.74). This anticorrelation remains high (− 0.73) also when the eastern basin strengths are related to the whole basin surface K.E. averaged over each timeslot.

We hypothesize that the amount of K.E. associated with semi-permanent jets, currents or large mesoscale eddies, grouped here together and named *KE fronts*, can be used as an indicator of their role as connectivity modulators. We identify *KE fronts* applying a pattern recognition algorithm on the K.E. fields for each time slot. The resulting pictures are processed by an image segmentation technique, based on K-means clustering, to separate the K.E. in four clusters of increasing energy content. The maximum-intensity group is selected as indicators for *KE fronts* and the number of pixels contained in each cluster is counted and used to estimate the size or abundance of each one. The maximum-intensity cluster well represents the energy-containing structures as measured by the correlation between the mean surface K.E. content in each time slot and the pixels within the corresponding cluster (c.c. > 0.99). The more pixels reside within each cluster, the larger the *KE fronts*-populated areas that this cluster approximates. This estimation is carried out for the whole basin, and separately in the eastern and western parts. The number of pixels is then correlated to the number of inferred ecoregions for the whole Mediterranean (c.c. = 0.81), and for eastern (c.c. = 0.81) and western (c.c. = 0.69) basins. Figure S6 in the *Supplementary Information* compares the clustering maps of a low energy time slot (1987–1993, in panel (a)) and a higher one (2004–2010 in panel (b)), for the whole Mediterranean Sea for the maximum cluster. The number of ecoregions is highly correlated with the *KE fronts* everywhere and especially in the eastern Mediterranean Sea. The higher level of fragmentation found in the *MAX* period is thus associated with more abundant and/or larger surface *KE fronts*, acting as eco-dynamical barriers.

To further strengthen this assessment, we consider that energy fronts can act as modulators for SSTa-derived domains. The ecoregionalization over a certain time slot characterizes that time range in one single ecoregion-map but stems from data known at several time points (i.e. monthly SSTa in our case). The resulting domains account therefore for the inherent physical variability of the system over time. A higher (lower) ecoregions fragmentation may therefore by associated with dynamical fronts occurring at different times and not necessarily in the same place, over a certain time range. If this is plausible, we expect to count more (less) occurrences of higher energy in broad areas where the domains are more (less) fragmented. For each time slot, the number of occurrences of a front in each pixel is therefore counted. Specifically, having defined a front as a K.E. realization above the 50th percentile of the overall (1987–2017) time varying surface K.E., we count how many times a front appears in the considered time slot at each pixel. In Figure S7 pixels are colored according to the number of occurrences in each time slot. The result is consistent with the domain fragmentation evolution. The higher fragmentation occurring in timeslots from 2001 to 2010 in the eastern basin is associated with more frequent fronts. Similar considerations hold for the other sub-basins, clearly distinguishing low energy periods from higher ones.

### Mediterranean ecoregions connectivity networks

Changes in functional networks or connectivity among ecoregions can be assessed by comparing a network from each energy period (*UP*: 1987–1993, *MAX*: 2004–2010 and *DOWN*: 2011–2017) (Fig. 3b,d,f for the eastern basin and Figure S8 in the *Supplementary Information* for the western basin).

In 1987–1993 the western basin was characterized by a high mean positive correlation of 0.73, with a strong, non-directional connectivity among the Tyrrhenian and Ligurian-Algero Provençal domains. In 2004–2010 the connectivity was overall weaker, and in particular reduced among Tyrrhenian waters. The connectivity between the Balearic domain (Bal) and the Tyrrhenian ones was also reduced. In 2011–2017 the connectivity was mostly recovered, especially in Tyrrhenian waters. In this period, the Algero-Provençal domain separated from the Ligurian Sea (Lig), enforcing its connectivity with the Balearic and the Alboran ecoregions.

In the eastern basin we focus our attention on the ecoregion immediately offshore the Suez Canal (Fig. 3), the major anthropogenic corridor for the introduction of non-indigenous marine species in the Mediterranean Sea, the so-called Lessepsian immigrants^32^. According to *δ*-MAPS, connectivity from the domain surrounding Suez was high in the first decade, decreased approaching *MAX*, remained small until about 2010–2011 with fewer statistically significant links, and increased again in the more recent time slot considered. During the *UP* and *DOWN* periods, the strongest connections were with the eastern Levantine (domain N), followed by that with the Aegean, Ionian and Tunisian Seas. During *UP* the connectivity extended to the Provençal and Algerian Seas, in the western basin, while in *DOWN* these links were absent and replaced by a connection with the Adriatic Sea.

The 1987–1993 and 2011–2017 periods, while not too dissimilar in energy levels, differed indeed for the phase of the Ionian-Adriatic Bimodal Oscillating System or BiOS^33,34^. The BiOS is a mode of variability characterized by a decadal reversal of the Northern Ionian Gyre (NIG) from cyclonic to anticyclonic, and vice versa. In its anticyclonic spinning the NIG deviates the inflowing Modified Atlantic Water (MAW) from the Sicily Channel towards the northern Ionian, entering the Adriatic Sea and decreasing its salinity and temperature. This prevents a portion of the MAW from reaching the Levantine basin, and enhances the outflow of Levantine waters into the western basin, along a pathway that follows the African coastline. The anticyclonic NIG co-occurs with higher concentrations of Atlantic and Western Mediterranean organisms in the Adriatic Sea. When the NIG is cyclonic, on the other hand, Levantine waters enter the Adriatic Sea, whereas the MAW preferably flows toward the Levantine^35^ and Lessepsian migrations influence the Adriatic Sea at various latitudes, affecting also phytoplankton phenology^33,36,37^. The corresponding regions and connectivity networks in the two opposite NIG periods are detailed in Figure S9 in the *Supplementary Information*.

## Discussion

### The Lessepsian invasion in the Mediterranean Sea

The investigation of the Mediterranean Sea ecoregions in the past 30 years revealed connectivity pathways of Lessepsian invasion (Fig. 3) that match, in the time averaged sense, the biodiversity patterns identified through the analysis of available coastal data, irrespective of time (Ref.^31^, their Fig. 2). Our analysis adds to the sparse coastal observations both space distribution and time variability of connectivity pathways, and helps contextualizing the timeline of the most recent, invasive, immigration into the Mediterranean, that of the lionfish or *Pterois miles*^32^. A first specimen was observed in 1991 off the coast of Israel^38^ but no other individuals were observed until 2012, when two lionfish were caught along the Lebanon coast. Spreading to other parts of the eastern Mediterranean then followed quickly, with specimens collected around Cyprus, Greece, Syria and Turkey by 2014, followed by Tunisia in 2015 and Italy in 2016 (Ref.^39^ and refs therein). The first immigration followed the pathway indicated by the strongest link in the 1987–1993 period from the Suez domain: Lw to N. It is likely that SST conditions limited spreading around 1990, because spawning in lionfish occurs preferentially when near surface water temperatures are at or above 28.4 °C. In the following decade the SSTs reached the favorable range during summer in most of the east Mediterranean, but the isolation of the Suez domain halted the invasion. With the shift to higher connectivity and the re-establishing of currents favoring transport across the east Mediterranean, lionfish easily spread after 2011, reaching as far as the coastline of Italy, with a timeline in agreement with the strength ordering identified by the network analysis.

In reference to the BiOS, we have shown that the anticyclonic to cyclonic transition is characterized by a shift towards positive correlations along Levantine waters and the Adriatic Sea. Periods of cyclonic NIG therefore may favor Lessepsian migrations into the Adriatic as outcome of the established connectivity patterns, while the anticyclonic phase favors Atlantic migration events due to the MAW intrusion. Species invasion in the Adriatic Sea has been explained through circulation changes, temperature rise and by the northward displacement or enlargement of thermophilic organisms’ habitat, from the southernmost areas of the Mediterranean basin towards more temperate regions. While some species spawning and settling may be favored moving through the time considered by the increasing seawater temperatures, our framework highlights how periodic circulation changes have been modulating the connectivity of the Ionian-Adriatic system all along. This cyclic behavior in invasion likelihood could and should be exploited by restoration efforts.

Biological invasions are a pervasive environmental problem in the Mediterranean Sea as they relate to the conservation of its biodiversity. They have been the focus of intense research over the past two decades, yet accurate predictions of spreading pathways and regional susceptibility remain elusive. Here we have shown that key information about the spatial extent, directionality and time variability of these invasions can be added through complex network analysis applied to sea surface temperature fields routinely available at low computational cost, opening new possibilities for their management and containment.

### Significance of proposed framework for marine ecoregionalization

A sustainable use of the oceans and their resources requires, among other actions, the protection and restoration of portions of the world coastlines and their ecosystems, and the conservation and restoration of marine biodiversity^40,41^. To achieve this goal, the identification of ecoregions and their connectivity in time and space is key to developing effective strategies for targeted assessments, environmental management, and implementation of marine protected areas. By exploiting the relationship between sea surface temperature anomalies and ocean currents that spans time and space scales of relevance to the sustainable development of marine ecosystems, we introduced an ecoregionalization framework based on connectivity. The framework builds on δ-MAPS, a complex network analysis tool related to multivariate statistics, clustering and community detection. δ-MAPS identifies the semi-autonomous components of the field under investigation, in our case sea surface temperature anomalies, and their (potentially) lagged and weighted interrelationships. It has been applied to the Mediterranean Sea for validation purposes, but is applicable to all oceans at mid-, tropical and equatorial latitudes. It provides key information regarding physical barriers to dispersion of larvae and juveniles, and the likelihood of spread among ecoregions over time. δ-MAPS is fast and automated, and it can be combined and augmented with the analysis of surface temperature and chlorophyll trends, both fields being available through satellite observations and constantly upgraded, quality controlled, data assimilation products. In the ocean, surface temperatures are directly linked to oxygen and CO_2_ content, while chlorophyll connects to nutrient availability, planktonic distributions and eutrophication episodes. δ-MAPS can also be applied to model simulations and future ocean projections whenever the resolution is high enough to resolve mesoscale circulations.

Ecoregions are essential units of comparative analysis in the assessment, management and solution of ecosystems problems. In the oceans, ecoregionalization is an interdisciplinary endeavor that involves physical, biological and ecological oceanographers, complicated, in comparison to land systems, by the presence of ocean currents connecting far away water bodies. δ-MAPS, through complex network analysis of a routinely observed quantity, SST, introduces a new, powerful tool to evaluate the physical contribution and its changes over time in most of the global Ocean.

## Supplementary Information

Supplementary Information

## Data Availability

The datasets analyzed are from the Mediterranean Sea Physical Reanalysis CMEMS MED-Physics^25^ (Simoncelli, S. et al. Mediterranean Sea physical reanalysis (MEDREA 1987–2015) (Version 1). Copernic. Monit. Environ. Mar. Serv. CMEMS (2014), https://doi.org/10.25423/medsea_reanalysis_phys_006_004). The δ-MAPS software can be found at https://github.com/FabriFalasca/delta-MAPS.

## References

[1] Loveland TR, Merchant JM (2004). Ecoregions and ecoregionalization: Geographical and ecological perspectives. Environ. Manage..

[2] Berline L, Rammou A-M, Doglioli A, Molcard A, Petrenko A (2014). A connectivity-based eco-regionalization method of the Mediterranean Sea. PLoS ONE.

[3] Giakoumi S (2013). Ecoregion-based conservation planning in the mediterranean: Dealing with large-scale heterogeneity. PLoS ONE.

[4] Kritzer JP, Sale PF (2004). Metapopulation ecology in the sea: from Levins’ model to marine ecology and fisheries science. Fish Fish..

[5] Forbes, E. *Map of the distribution of marine life. Pages 99–102 and plate 131 in Johnston AK. ed The Physical Atlas of Natural Phenomena*. (William Blackwood and Sons, 1856).

[6] Briggs, J. *Global Biogeography*. (Elsevier, 1995).

[7] Ekman, S. *Zoogeography of the Sea*. (Sidgwick & Jackson, 1953).

[8] Longhurst, A. Seasonal cycles of pelagic production and consumption. *Prog. Oceanogr.* **36**, 77–167 (1995).

[9] Oliver, M. J. & Irwin, A. J. Objective global ocean biogeographic provinces. *Geophys. Res. Lett.* **35**, 1–6 (2008).

[10] D’Ortenzio, F. On the trophic regimes of the Mediterranean Sea. 25 (2008).

[11] Koubbi, P. *et al.* Ecoregionalization of myctophid fish in the Indian sector of the Southern Ocean: Results from generalized dissimilarity models. *Deep Sea Res. Part II Top. Stud. Oceanogr.* **58**, 170–180 (2011).

[12] Casale P, Mariani P (2014). The first ‘lost year’ of Mediterranean sea turtles: Dispersal patterns indicate subregional management units for conservation. Mar. Ecol. Prog. Ser..

[13] Rossi V, Ser-Giacomi E, López C, Hernández-García E (2014). Hydrodynamic provinces and oceanic connectivity from a transport network help designing marine reserves. Geophys. Res. Lett..

[14] Ser-Giacomi E, Rossi V, López C, Hernández-García E (2015). Flow networks: A characterization of geophysical fluid transport. Chaos Interdiscip. J. Nonlinear Sci..

[15] Ser-Giacomi, E. *et al.* Impact of climate change on surface stirring and transport in the Mediterranean Sea. *Geophys. Res. Lett.* **47**, e2020GL089941. 10.1029/2020GL089941 (2020).

[16] Rosvall M, Bergstrom CT (2008). Maps of random walks on complex networks reveal community structure. Proc. Natl. Acad. Sci..

[17] Levins, R. Some demographic and genetic consequences of environmental heterogeneity for biological control. *Bull. Entomol. Soc. Am.* **15**(3), 237–240 10.1093/besa/15.3.237 (1969).

[18] Obura D (2012). The diversity and biogeography of Western Indian Ocean reef-building corals. PLoS ONE.

[19] Thompson DM (2018). Variability in oceanographic barriers to coral larval dispersal: Do currents shape biodiversity?. Prog. Oceanogr..

[20] Ciavatta S (2019). Ecoregions in the mediterranean sea through the reanalysis of phytoplankton functional types and carbon fluxes. J. Geophys. Res. Oceans.

[21] Crochelet E (2016). A model-based assessment of reef larvae dispersal in the Western Indian Ocean reveals regional connectivity patterns—Potential implications for conservation policies. Reg. Stud. Mar. Sci..

[22] Fountalis I, Dovrolis C, Bracco A, Dilkina B, Keilholz S (2018). δ-MAPS: from spatio-temporal data to a weighted and lagged network between functional domains. Appl. Netw. Sci..

[23] Falasca F, Bracco A, Nenes A, Fountalis I (2019). Dimensionality reduction and network inference for climate data using *δ*-MAPS: Application to the CESM large ensemble sea surface temperature. J. Adv. Model. Earth Syst..

[24] Leeuwenburgh O, Stammer D (2001). The effect of ocean currents on sea surface temperature anomalies. J. Phys. Oceanogr..

[25] Simoncelli, S. *et al.* Mediterranean Sea physical reanalysis (MEDREA 1987–2015) (Version 1). *Copernic. Monit. Environ. Mar. Serv. CMEMS* (2014). 10.25423/medsea_reanalysis_phys_006_004.

[26] Saiz, E., Sabatés, A. & Gili, J.-M. The Zooplankton. in *The Mediterranean Sea* (eds. Goffredo, S. & Dubinsky, Z.) 183–211 (Springer Netherlands, 2014). 10.1007/978-94-007-6704-1_11.

[27] Bianchi CN, Morri C (2000). Marine biodiversity of the mediterranean sea: Situation, problems and prospects for future research. Mar. Pollut. Bull..

[28] Coll M (2010). The biodiversity of the Mediterranean Sea: estimates, patterns, and threats. PLoS ONE.

[29] Pinardi, N., Zavatarelli, M. & Arneri, E. The physical, sedimentary and ecological structure and variability of shelf areas in the Mediterranean Sea (27,S). 30.

[30] Bas, C. The Mediterranean: A synoptic overview. *Contrib. Sci.* 25–39 (2009). 10.2436/20.7010.01.57.

[31] Katsanevakis, S. *et al.* Invading the Mediterranean Sea: biodiversity patterns shaped by human activities. *Front. Mar. Sci.* **1**, (2014).

[32] Bariche M, Kleitou P, Kalogirou S, Bernardi G (2017). Genetics reveal the identity and origin of the lionfish invasion in the Mediterranean Sea. Sci. Rep..

[33] Civitarese G, Gačić M, Lipizer M, Borzelli GLE (2010). On the impact of the Bimodal Oscillating System (BiOS) on the biogeochemistry and biology of the Adriatic and Ionian Seas (Eastern Mediterranean). Biogeosci. Discuss..

[34] Rubino A (2020). Experimental evidence of long-term oceanic circulation reversals without wind influence in the North Ionian Sea. Sci. Rep..

[35] Menna, M. *et al.* Decadal variations of circulation in the Central Mediterranean and its interactions with mesoscale gyres. *Deep Sea Res. Part II Top. Stud. Oceanogr.* **164**, 14–24 (2019).

[36] Batistić M, Garić R, Molinero J (2014). Interannual variations in Adriatic Sea zooplankton mirror shifts in circulation regimes in the Ionian Sea. Clim. Res..

[37] Lavigne H, Civitarese G, Gačić M, D’Ortenzio F (2018). Impact of decadal reversals of the north Ionian circulation on phytoplankton phenology. Biogeosciences.

[38] Golani D, Sonin O (1992). New records of the Red Sea fishes, Pterois miles (Scorpaenidae) and Pteragogus pelycus (Labridae) from the eastern Mediterranean Sea. Jap. J. Ichthyol..

[39] Savva I (2020). They are here to stay: the biology and ecology of lionfish ( *Pterois miles* ) in the Mediterranean Sea. J. Fish Biol..

[40] IOC-Unesco (2018b). Roadmap for the UN Decade of Ocean Science for Sustainable Development, Version 2.0.

[41] Visbeck M (2018). Ocean science research is key for a sustainable future. Nat. Commun..

[42] Box, G. E., Jenkins, G. M. & Reinsel, G. C. *Time series analysis: forecasting and control.* (Wiley, 2011).

