# Supplementary material for: Uncovering marine connectivity through sea surface temperature
Source: Sci Rep. 2021 Apr 23;11:8839. doi: 10.1038/s41598-021-87711-z (PMC8065169; doi:10.1038/s41598-021-87711-z)
Supplement: Supplementary file 1 — Supplementary Information [file 41598_2021_87711_MOESM1_ESM.docx]

### **Supplementary Information.**


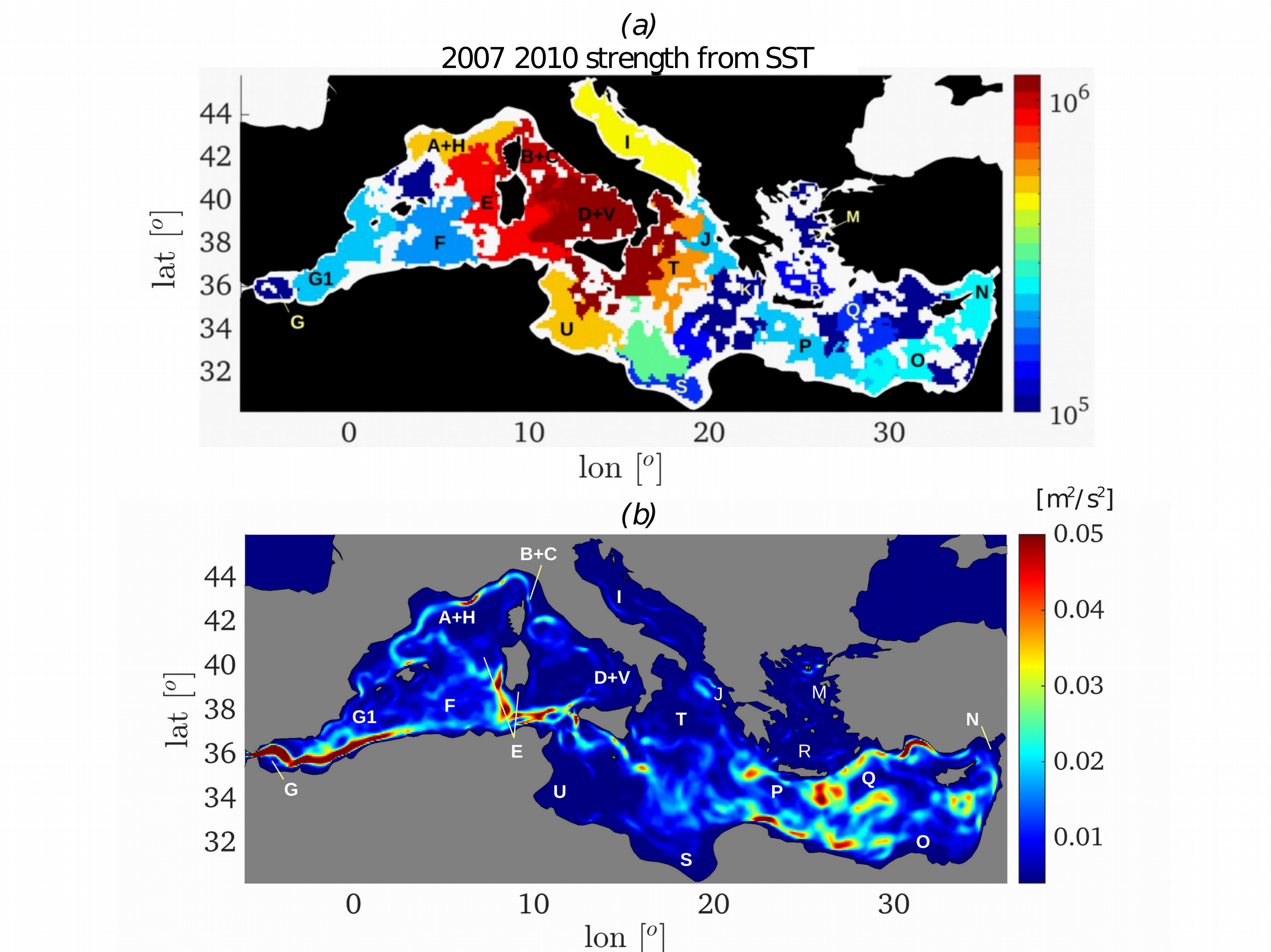


**Fig. S1.** (a) domains and the corresponding strengths (in color) for the validation period 2007-2010. Ecoregion (domain) labels are as in (Berline et al. 2014) to ease comparison. (b) 2007-2010 averaged surface kinetic energy per unit mass. The most energetic structures act as transport barriers. This can be qualitatively seen comparing the two plots, as strong currents largely bound the domains, which are also indicated in this panel (white letter labels). (Visualization produced with Matlab R2018a, <https://www.mathworks.com/>)


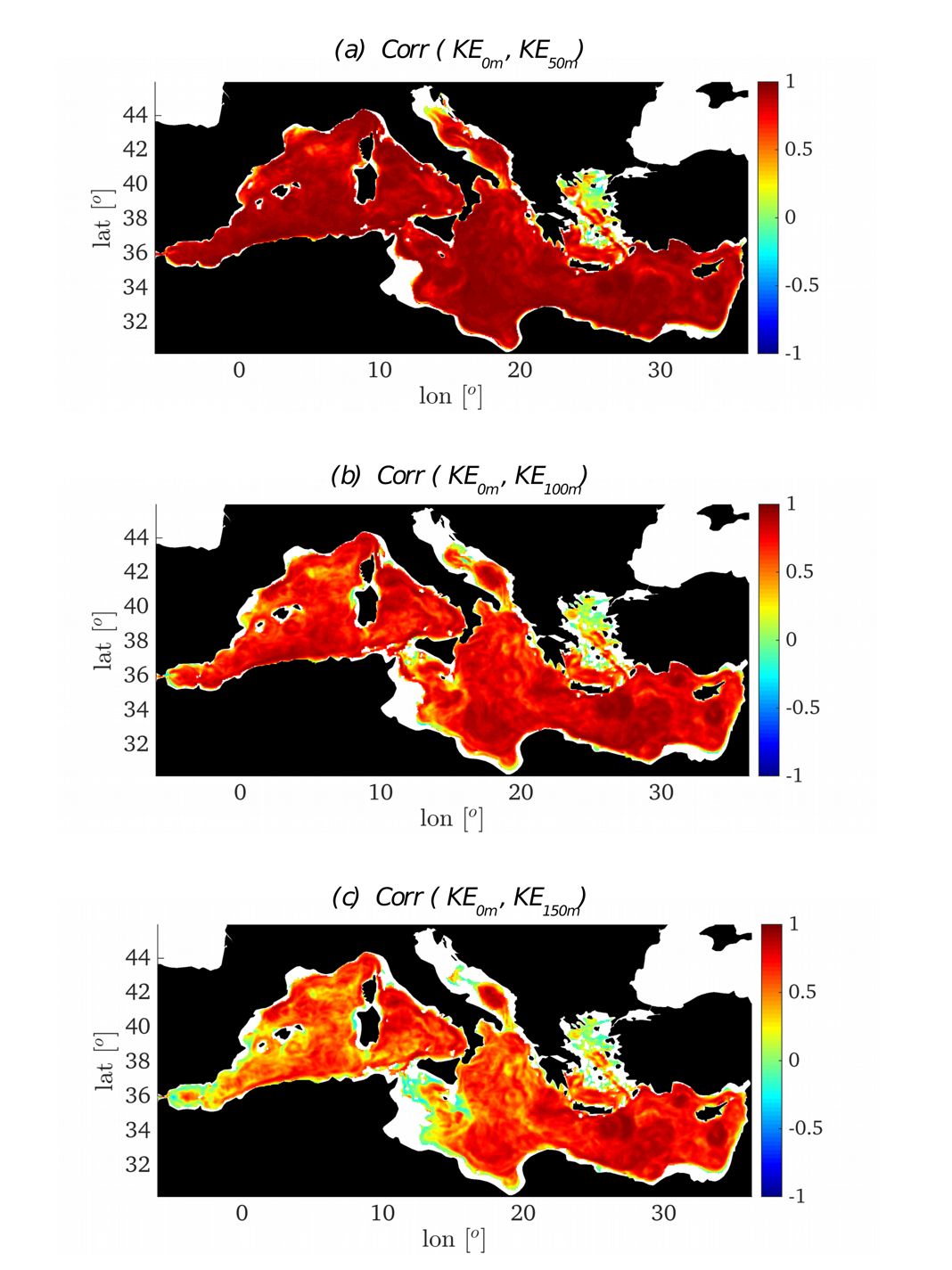


**Fig. S2.** 1987-2017 correlation maps between the kinetic energy per unit mass at the ocean surface and that at 50 m (a), 100 m (b), and 150 m depth (c). The kinetic energy is obtained from the velocity components (zonal and meridional) retrieved by the CMEMS MED Physics reanalysis. (Visualization produced with Matlab R2018a, <https://www.mathworks.com/>)


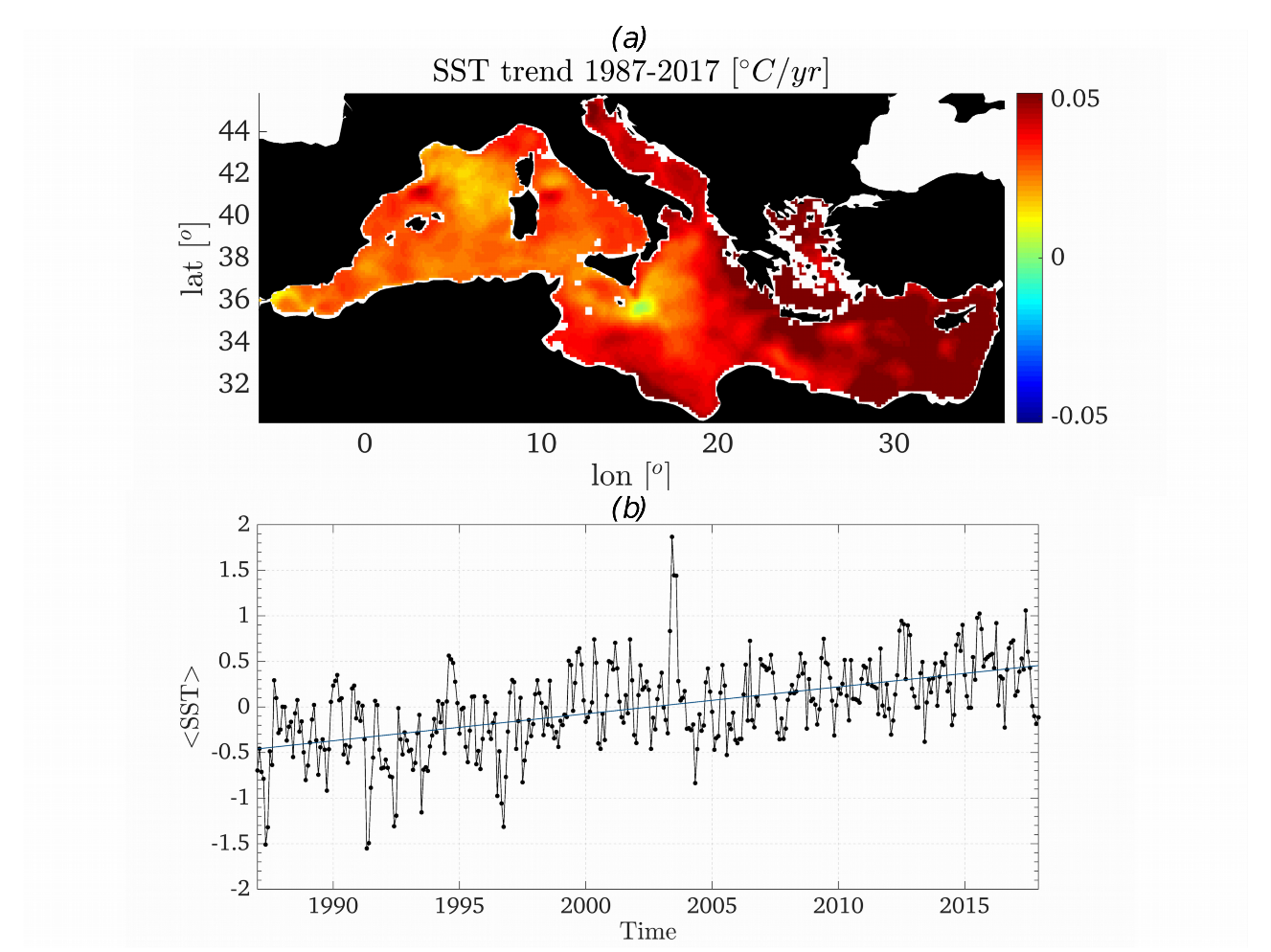


**Fig. S3.** (a): sea surface temperature (SST) trend over 1987-2017 for the entire Mediterranean Sea.
(b): time evolution of the SST anomalies (°C) averaged over the basin. The linear trend is indicated as a blue line. (Visualization produced with Matlab R2018a, <https://www.mathworks.com/>)


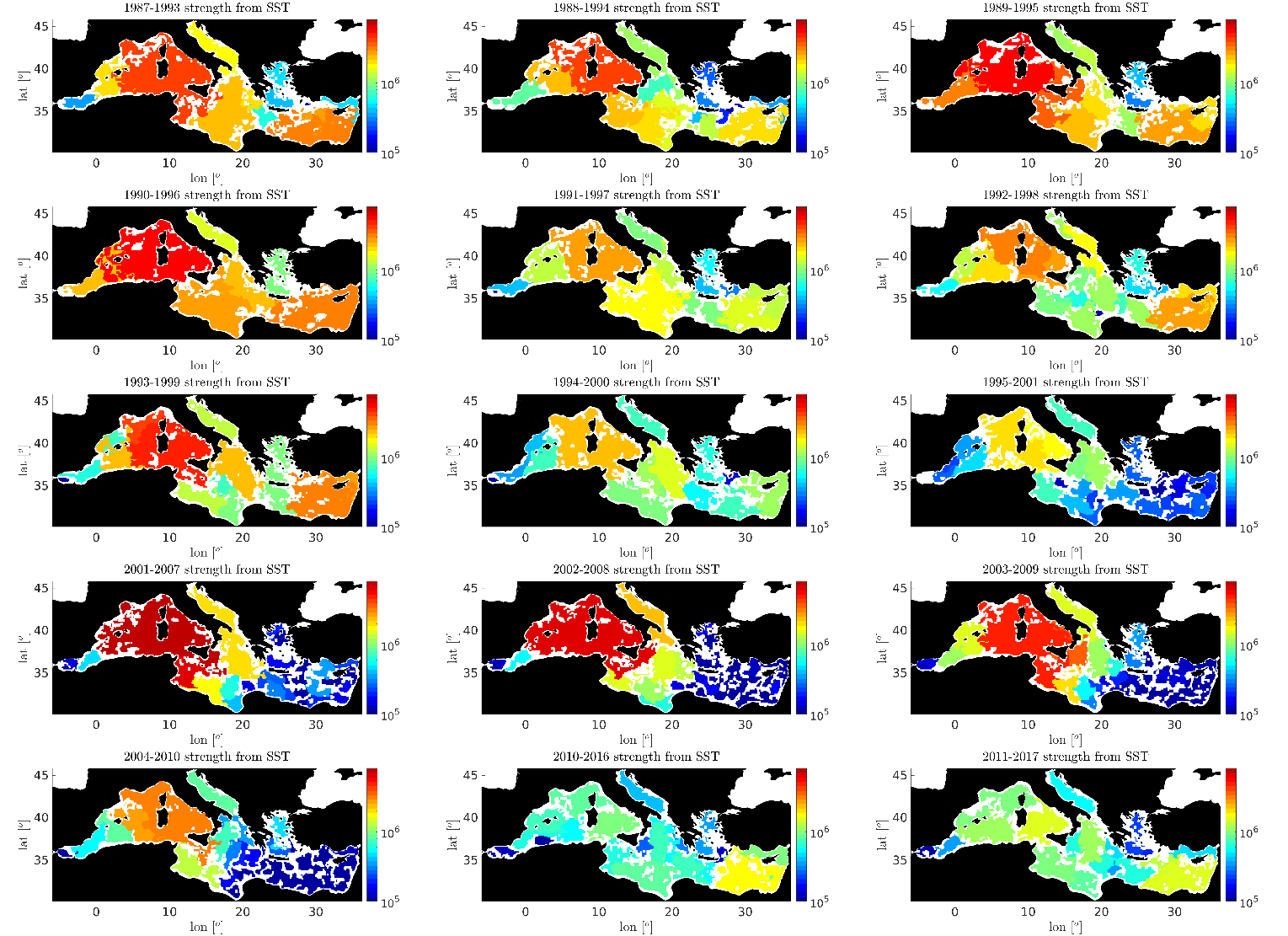


**Fig. S4.** Domain strength maps for all 7-years time slots. In the UP interval, the domain strengths are nearly homogeneously distributed among the western and eastern basin. Two well-connected overlapping ecoregions occupy most of the Levantine Sea, the Ionian and Aegean Seas are clearly separated, and the Adriatic Sea is enclosed by a single domain, a characteristic that does not change over time. Macro-areas occupy the western Mediterranean basin with large overlaps in the Ligurian, Algero-Provençal and Tyrrhenian Seas, and domains with lower strengths are found in the Alboran and Balearic Seas. A transition phase occurs by the end of UP, namely from 1994 to 2001, when the kinetic energy approaches its maximum values, with an overall weakening of domains, especially in the eastern basin. In the MAX time slot (2004-2010) the ecoregions from the Ionian to the Levantine Seas undergo spatial fragmentation, separation and further weakening, while the Adriatic and the Aegean domains retain size and strength. Changes across time slots are generally insignificant during MAX, except for a slight increase in fragmentation in the western basin. Finally, the DOWN time slots are characterized by size and strength recovery in the eastern basin, along with a weakening of the western domains, thus returning to an overall homogeneous strength distribution, marked by lower strengths compared to the initial state as the overall kinetic energy remains higher. (Visualization produced with Matlab R2018a, <https://www.mathworks.com/>)


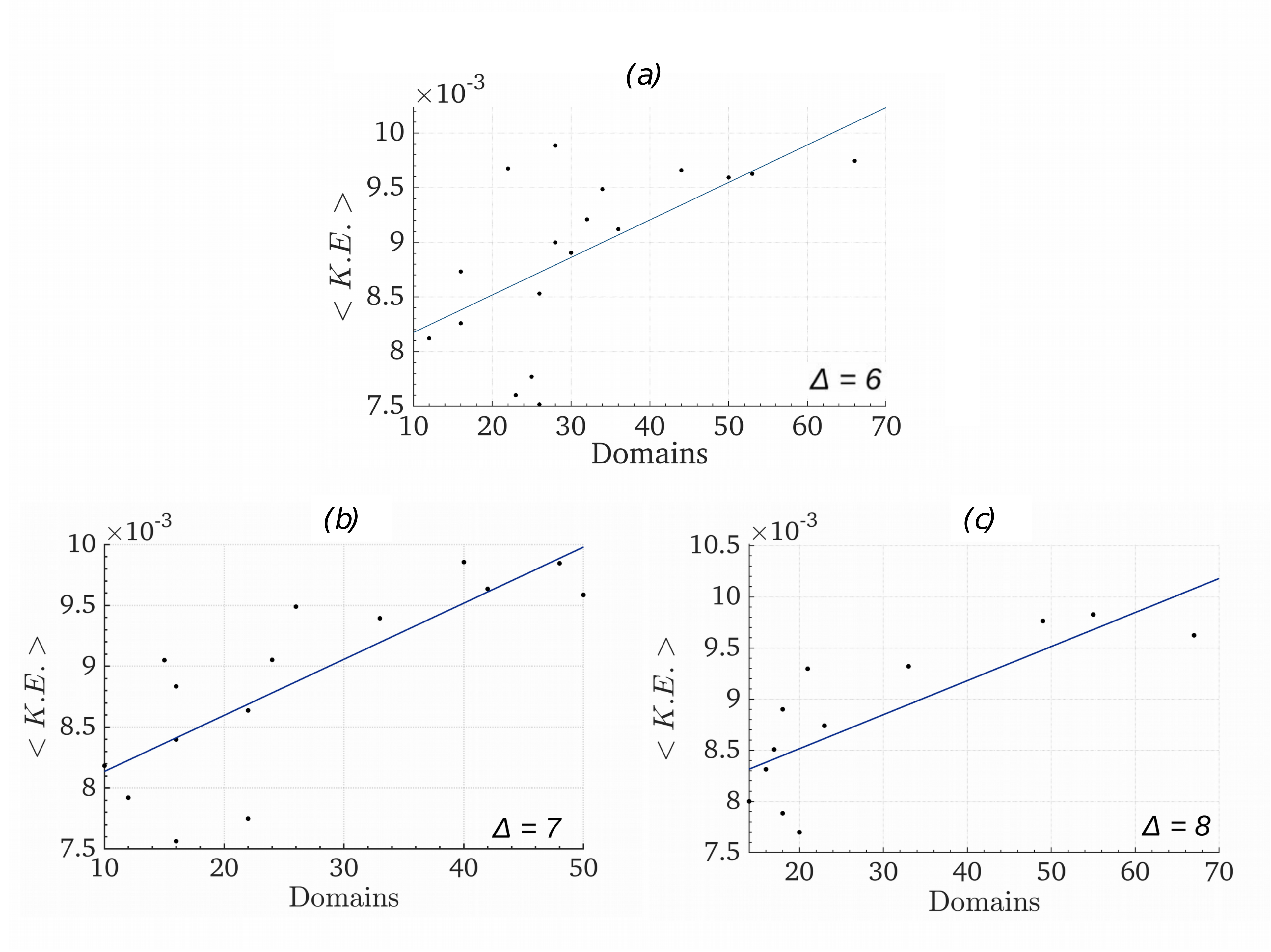


**Fig. S5.** We assessed that the link between the ecoregionalization framework and the upper ocean energetics is robust to changes of the time slot length. All possible combinations with Δ = 6,7,8 years covering the whole period 1987-2017 have been verified, excluding overlaps by more than one year among different trends periods. Positive correlations between the number of ecoregions and surface kinetic energy content are found in each case, with better results for Δ = 7 or 8 years. The figure shows the scatter plots of the number of ecoregions (horizontal axis) plotted against the surface kinetic energy (per unit mass) average content (m^2^/s^2^) in each time slot, for (a) Δ=6 year, (b) 7 years, and (c) and 8 years. The linear regression fit is shown in each panel as blue line. Pearson’s correlation coefficients are 0.61, 0.79 and 0.79 for Δ = 6,7,8 years, respectively.


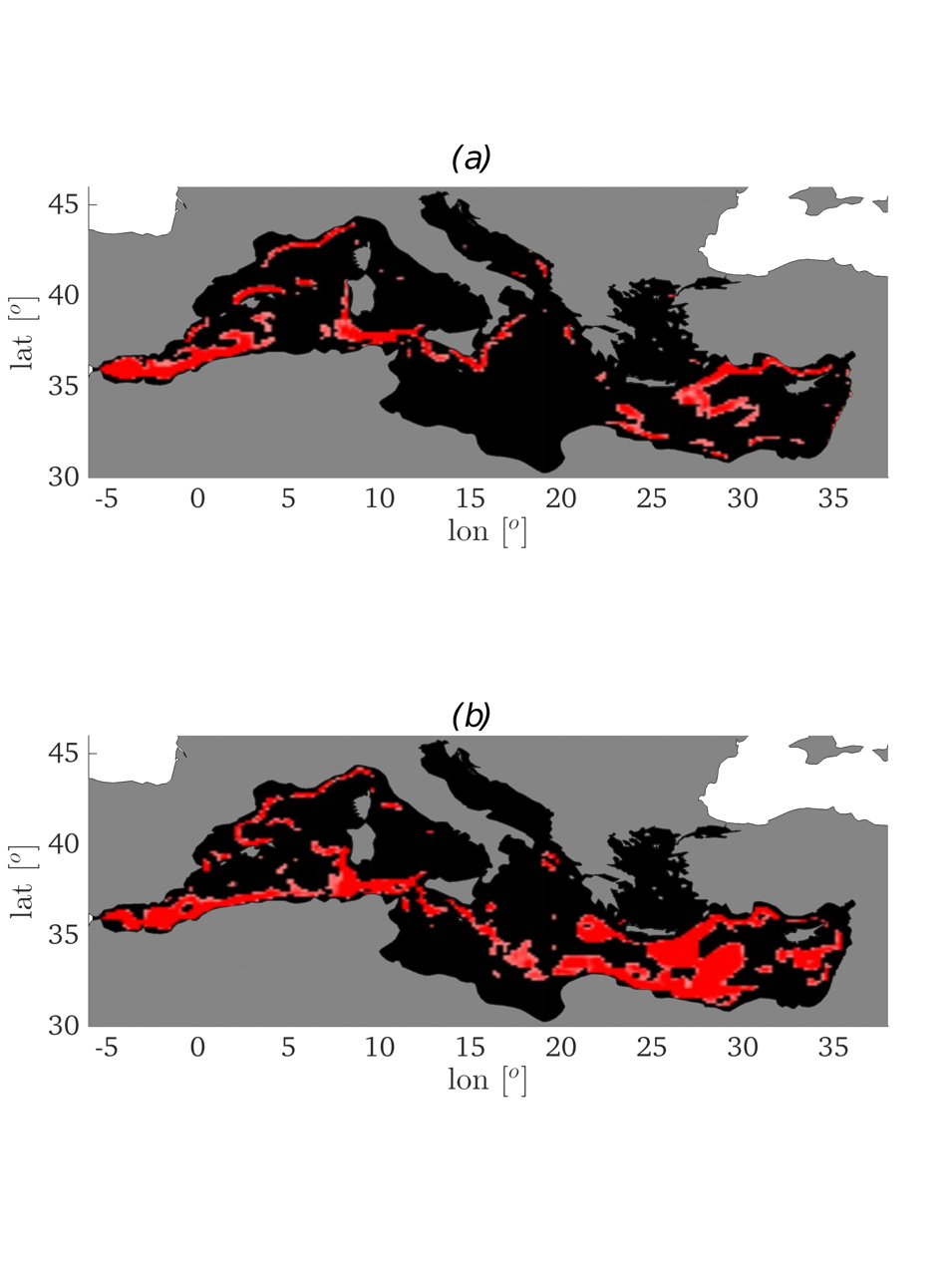


**Fig. S6.** Energy containing maximum cluster resulting from the pattern recognition K-means algorithm, for a less energetic and less fragmented time slot (1987-1993 in panel (a)), and a more energetic and more fragmented one (2004-2010 in panel (b)). (Visualization produced with Matlab R2018a, <https://www.mathworks.com/>)


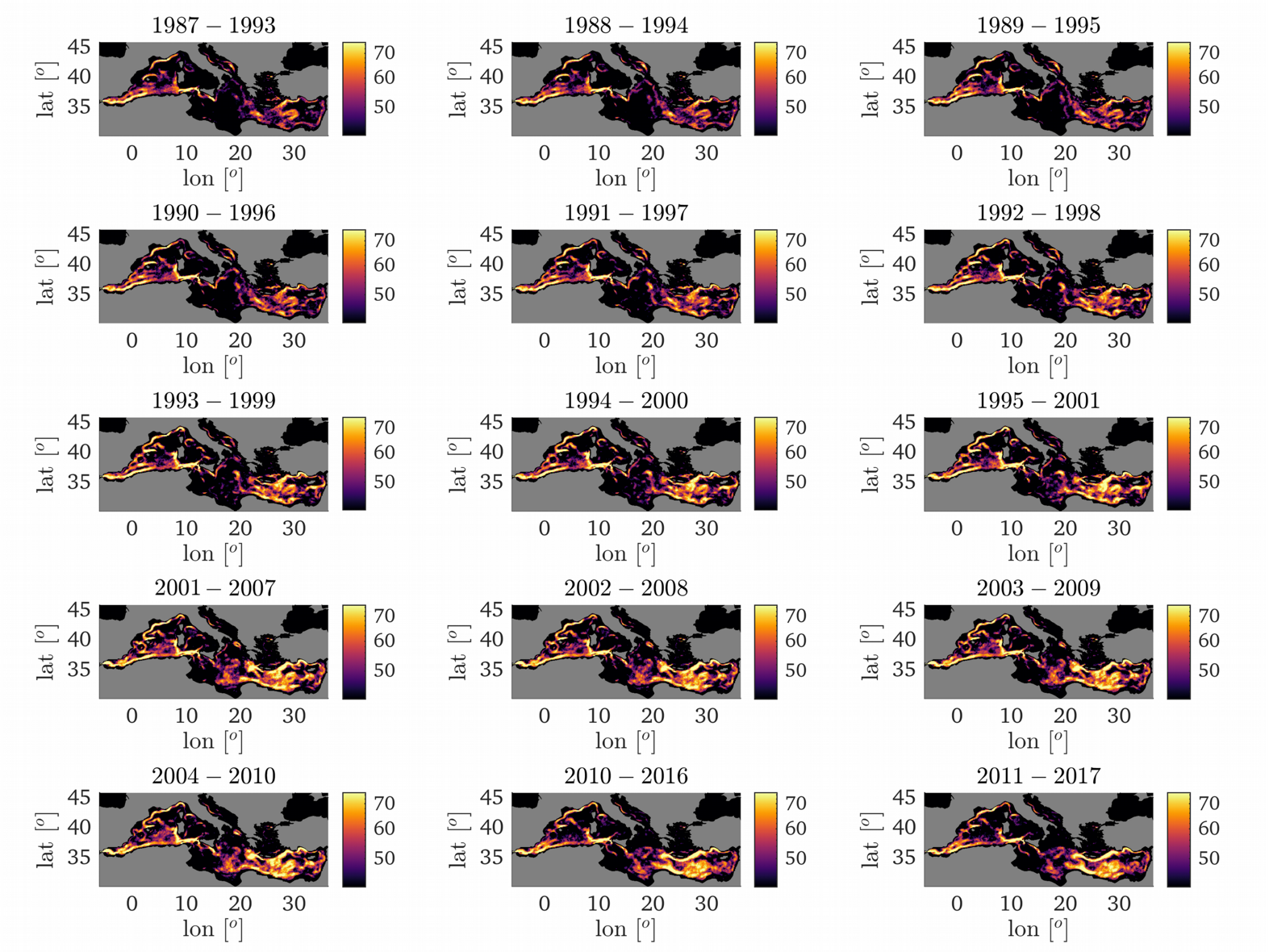


**Fig. S7. Occurrences of surface K.E. fronts for all 7-years time slots**. In each panel (i.e. time slot), the number of occurrences of a front in each pixel is reported. A front is defined as the value of surface k.e. above the 50th percentile of the overall (1987-2017) time varying surface K.E. Pixels where a front has passed, at any time, are colored according to the number of occurrences. Values occurring less than the average are shown as darker areas, whereas more frequently populated areas are colored as indicated. (Visualization produced with Matlab R2018a, <https://www.mathworks.com/>)


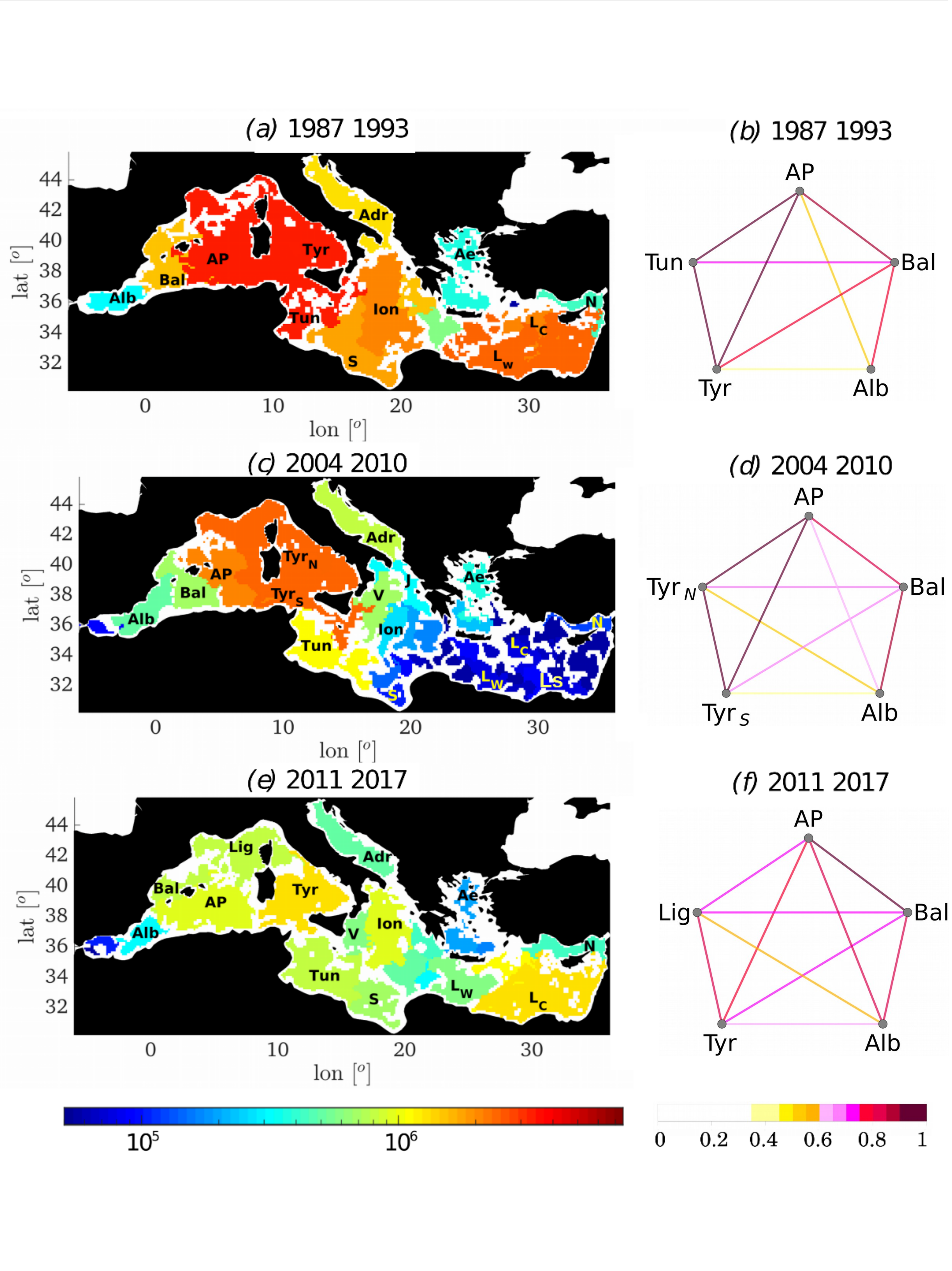


**Fig. S8.** Domains colored by their strength for the whole Mediterranean Sea (left) and connectivity networks for the western basin (right) for the three 7-years timeslots selected as representative of the UP (panels (a),(b)), MAX (panels (c),(d)) and DOWN (panels (e),(f)) periods. The color of the links in the connectivity nets indicates the correlation between domains, as labeled. Only correlations stronger than 0.35 are plotted. (Domains maps visualization produced with Matlab R2018a, <https://www.mathworks.com/>)


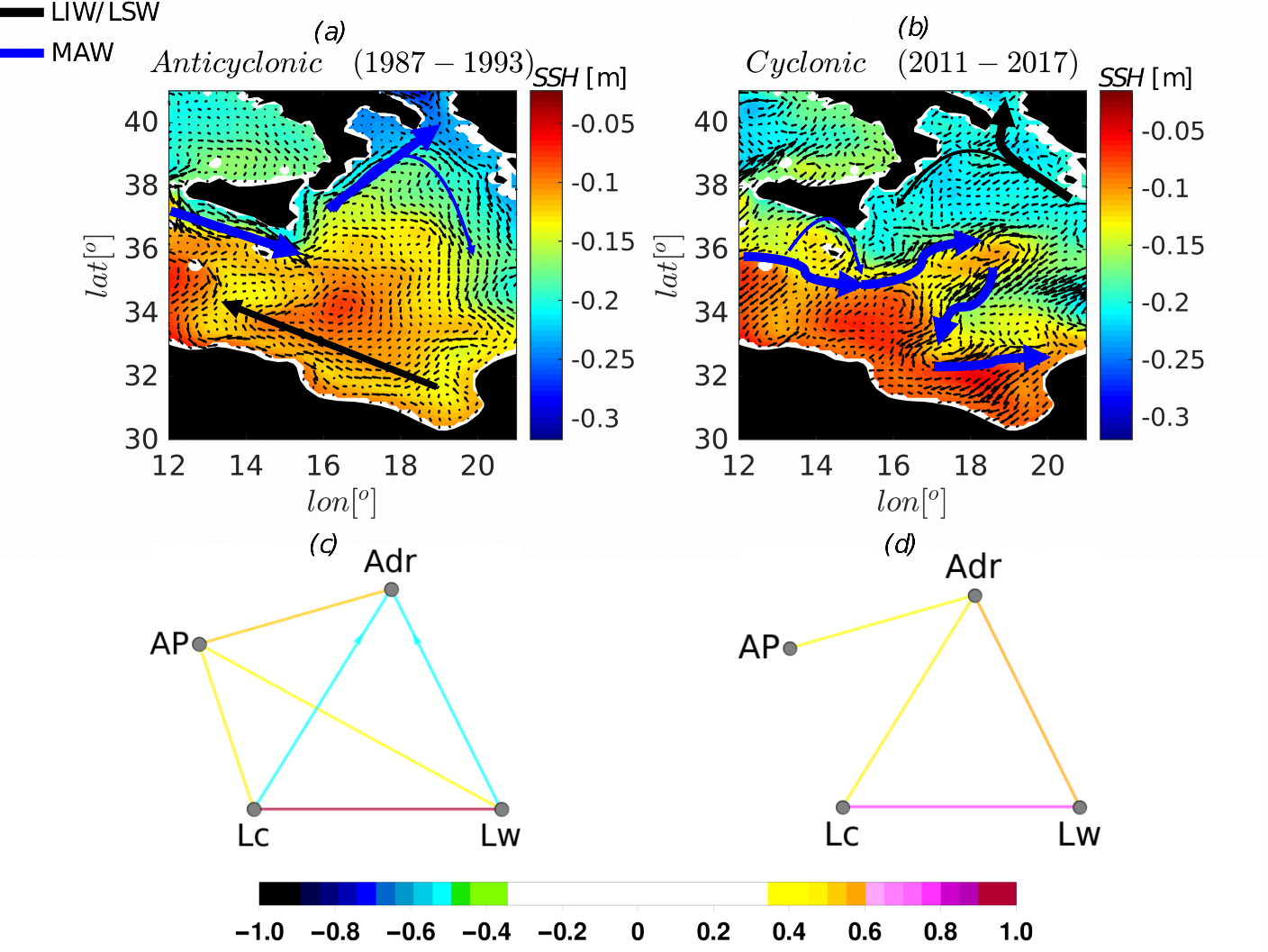


**Fig. S9.** A schematic of the MAW and Levantine waters pathway during the anticyclonic (a) and cyclonic (b) NIG phases of the BiOS with the corresponding relevant connectivity networks in panel (c) and (d), respectively. Changes in connectivity between the anticyclonic and cyclonic NIG modes are captured by the sign reversal of Levantine-Adriatic links (Lc-Adr and Lw-Adr), which switches from negative (anticyclonic NIG) to positive (cyclonic NIG). During the anticyclonic NIG, more MAW enters the Adriatic and the positive correlation among the western basin AP and the Adriatic Sea is stronger. In the cyclonic NIG phase, on the other hand, AP-Adr are weakly linked, consistently with a halted flow of MAW into the Adriatic. Moreover, during the cyclonic NIG the main path of (warmer and saltier) Levantine waters reaches the Adriatic basin increasing its temperature and salinity. This corresponds to a positive correlation between the LW main path temperature and salinity anomalies and those of the Adriatic Sea, as Levantine waters warm the Adriatic. During the anticyclonic NIG, the main path of (warmer and saltier) Levantine waters does not preferably flow into the Adriatic, which is reached instead by the cooler and fresher MAW. This is expressed by a negative correlation between the LW domain and its temperature anomalies and the Adriatic Sea. (Maps visualization produced with Matlab R2018a, <https://www.mathworks.com/>)
